# Urinary Volatile Organic Compound Metabolites Are Associated with Reduced Lung Function in U.S. Children and Adolescents

**DOI:** 10.3390/toxics12040289

**Published:** 2024-04-16

**Authors:** Angelico Mendy, Sara Burcham, Ashley L. Merianos, Tesfaye B. Mersha, Kimberly Yolton, Aimin Chen, E. Melinda Mahabee-Gittens

**Affiliations:** 1 Division of Epidemiology, Department of Environmental and Public Health Sciences, University of Cincinnati College of Medicine, Cincinnati, OH 45229, USA; angelico.mendy@uc.edu (A.M.); burchasa@mail.uc.edu (S.B.); 2 School of Human Services, University of Cincinnati, Cincinnati, OH 45221, USA; ashley.merianos@uc.edu; 3 Division of Asthma Research, Cincinnati Children’s Hospital Medical Center, University of Cincinnati College of Medicine, Cincinnati, OH 45229, USA; tesfaye.mersha@cchmc.org; 4 General and Community Pediatrics, Cincinnati Children’s Hospital Medical Center, University of Cincinnati College of Medicine, Cincinnati, OH 45229, USA; kimberly.yolton@cchmc.org; 5 Department of Biostatistics, Epidemiology and Informatics, Perelman School of Medicine, University of Pennsylvania, Philadelphia, PA 19104, USA; aimin.chen@pennmedicine.upenn.edu; 6 Division of Emergency Medicine, Cincinnati Children’s Hospital Medical Center, University of Cincinnati College of Medicine, Cincinnati, OH 45229, USA

**Keywords:** volatile organic compounds, environmental exposures, urinary metabolites, children respiratory health

## Abstract

(1) Background: Volatile organic compounds (VOCs) are indoor pollutants absorbed by inhalation. The association of several VOCs with lung function in children and adolescents is unknown. (2) Methods: We analyzed 505 participants, 6–17-year-olds from the 2011–2012 National Health and Nutrition Examination Survey. Multiple linear regression models were fitted to estimate the associations of VOC metabolites with spirometry outcomes adjusting for covariates. (3) Results: Urinary metabolites of xylene, acrylamide, acrolein, 1,3-butadiene, cyanide, toluene, 1-bromopropane, acrylonitrile, propylene oxide, styrene, ethylbenzene, and crotonaldehyde were all detected in ≥64.5% of participants. Forced expiratory volume in 1 s (FEV_1_) % predicted was lower in participants with higher levels of metabolites of acrylamide (β: −7.95, 95% CI: −13.69, −2.21) and styrene (β: −6.33, 95% CI: −11.60, −1.07), whereas the FEV_1_ to forced vital capacity (FVC) ratio % was lower in children with higher propylene oxide metabolite levels (β: −2.05, 95% CI: −3.49, −0.61). FEV_1_ % predicted was lower with higher crotonaldehyde metabolite levels only in overweight/obese participants (β: −15.42, 95% CI: −26.76, −4.08) (P_interaction_ < 0.001) and with higher 1-bromopropane metabolite levels only in those with serum cotinine > 1 ng/mL (β: −6.26, 95% CI: −9.69, −2.82) (P_interaction_ < 0.001). (4) Conclusions: We found novel associations of metabolites for acrylamide, propylene oxide, styrene, 1-bromopropane and crotonaldehyde with lower lung function in children and adolescents.

## 1. Introduction

Volatile organic compounds (VOCs) are carbon-containing chemicals capable of evaporating in the environment at room temperature [1]. VOCs are considered major indoor pollutants and can be found in various products such as paints, solvents, cleaning or personal care products, wall and floor covers, biomass burning emissions, furniture, and cigarette smoke [1]. Outdoor exposure to VOCs is also possible from sources such as automotive exhaust and traffic emissions, oil and gas industries, and background biogenic emissions [2]. VOCs enter the human body primarily through inhalation, and to a lesser extent, through dermal contact and oral ingestion; due to their volatility and lipophilicity, they are absorbed primarily in the lungs and mucous membranes [3]. Given the significant time people, and especially children, spend indoors, the potential health effects of these ubiquitous indoor pollutants are a cause for concern [4].

In animal models and in studies with high occupational exposures, VOCs are purportedly associated with adverse respiratory outcomes [5]. The mechanisms for these effects include the ability of these chemicals to cause airway and lung inflammation in humans through oxidative stress and inflammation as well as to trigger immunoglobulin E production by disturbing T-cell activity or increasing antigen-presenting activity [6]. Yet, few general population studies have investigated the adverse respiratory outcomes associated with exposure to VOCs and fewer studies have included children [7,8]. Most studies conducted in children performed indoor assessments of VOC exposure, and only three estimated personal exposure by assessing internal dose in relation to lung function [1]. Moreover, there have been no studies to date on the associations of exposure biomarkers of common VOCs such as acrylamide, acrolein, cyanide, butadiene, 1-bromopropane, propylene oxide, styrene, or crotonaldehyde with pulmonary function in children. Therefore, we aimed to assess the cross-sectional association of urinary VOC metabolites with pulmonary function and associated comorbidities among children and adolescents in a U.S. representative sample.

## 2. Methods

### 2.1. Data Source

We conducted an analysis from children and adolescents aged 6 to 17 years old who participated in the 2011–2012 National Health and Nutrition Examination Survey (NHANES). This survey was administered by the Centers for Disease Control and Prevention (CDC)’s National Center for Health Statistics (NCHS) to evaluate the health status of the non-institutionalized American civilian population [9]. NCHS recruited a U.S. representative sample through a complex multistage sampling design and obtained consent from all participants. The Institutional Review Boards (IRB) of the CDC and NCHS approved the NHANES protocols [9].

Of the 682 NHANES 2011–2012 participants aged 6–17 years old who had data on urinary VOCs, 634 underwent spirometry testing. After excluding participants with missing data on the nicotine metabolite serum cotinine (*n* = 99), body mass index (BMI) (*n* = 1), and household income (*n* = 38), the final sample used for analysis was 505 participants.

### 2.2. Urinary VOC Metabolites

VOC metabolites were measured in spot urine among participants ≥ 6 years old using ultra performance liquid chromatography coupled with electrospray tandem mass spectrometry. Our analysis included only urinary VOC metabolites with a detection frequency >50% and we imputed the samples that had levels below the limit of detection (LOD) with a value of LOD/√2. The VOC metabolites along with their abbreviations, parent compounds, as well as their LODs are reported in Table 1. Details on the laboratory methods and quality control are available at https://wwwn.cdc.gov/nchs/data/nhanes/2011-2012/labmethods/UVOC_G_MET_VOC_Metabolites.pdf (accessed on 5 April 2024) and have also been published elsewhere [10].

### 2.3. Spirometry

Spirometry was performed in the children and adolescents by trained technicians after a pretest screening questionnaire was administered to assess medical safety. Explanation and demonstration of spirometry procedures were provided to participants who then performed five to eight forced vital capacity (FVC) maneuvers that met the American Thoracic Society (ATS) acceptability and reproducibility criteria [11]. We defined lung function as the forced expiratory volume in 1 s (FEV_1_) to FVC ratio percent and we calculated FEV_1_ % predicted using the Hankinson equations that standardize for age, sex, race/ethnicity, and height [12]. FEV_1_ % predicted serves as a means for grading asthma severity and has been shown to be a strong predictor of asthma attacks in children [13].

### 2.4. Covariates

NCHS collected data on participants’ age, sex, race/ethnicity, and annual family income using questionnaires. The poverty income ratio (PIR) or the ratio of family income to poverty was estimated, adjusting for family size, year of survey, and state of residence [14]. BMI was calculated as weight (kilograms) over height (meters) squared and classified as <85th percentile (normal), ≥85th percentile to <95th percentile (overweight), and ≥95th percentile (obese), in accordance to the CDC guidelines for BMI categorization in children and adolescents [15]. We adjusted for urine dilution using urinary creatinine measured by quantitative enzymatic determination [15]. Serum cotinine, a widely used nicotine metabolite that has been associated with reduced lung function, was analyzed by an isotope dilution-high performance liquid chromatography coupled with atmospheric pressure chemical ionization tandem mass spectrometry [16].

### 2.5. Statistical Analysis

Descriptive analyses were performed to examine the distribution of creatinine uncorrected and corrected concentrations of urinary VOC metabolites (the ratio of VOC urinary metabolites levels over creatinine concentrations to account for urine dilution). *p*-values for comparing geometric means (GMs) of creatinine-corrected urinary VOC metabolites by study participants’ characteristics were estimated. We log transformed the creatinine uncorrected and corrected urinary VOC metabolites’ levels to improve the distribution normality due to their skewness. We assessed the intercorrelations between the log_10_-transformed levels of creatinine-corrected VOCs by means of spearman correlations. To estimate the association of urinary VOC metabolites with lung function, we used linear regression modeling in order to estimate regression coefficients (β) and their corresponding 95% confidence interval (CI), adjusting for age, height, PIR, log-cotinine and BMI used as continuous variables and for sex and race/ethnicity used as categorical variables. We corrected for urine dilution in regression analyses by including urinary creatinine as a separate independent variable, along with the creatinine-uncorrected VOC metabolites levels as recommended by Barr et al., so that the observed exposure-outcome associations are independent of the creatinine variable [15,17]. To test whether the association of urinary VOC metabolites with airflow obstruction differed with pre-existing asthma, serum cotinine or obesity, we assessed these variables for effect modification on a multiplicative scale by including interaction terms in the models. We performed these analyses in SAS (Version 9.4; SAS Institute, Cary, NC, USA), and we accounted for the NHANES sampling weights as well as the complex survey design, so that our estimates were nationally representative. *p*-values < 0.05 were set as statistically significant in all the analyses.

To flexibly assess the association between the mixtures of the VOC urinary metabolites with lung function, we performed Bayesian Kernel Machine regression (BKMR) models. These allow estimating the nonlinear and non-additive exposure-outcomes relationships and were adjusted for age, sex, height, race/ethnicity, PIR, log-cotinine, urinary creatinine and BMI. BKMR was performed in R Studio (Version 4.3.2; R Foundation, Vienna, Austria).

## 3. Results

### 3.1. Distribution of Urinary VOC Metabolites

Urinary VOC metabolites were detected in at least 64.5% of the 505 participants included in our analysis; the urinary metabolites of acrylamide, acrolein, 1,3-butadiene, xylene and crotonaldehyde were detected in 100% of the urine samples (Table 1). The GMs of creatinine-corrected urinary VOC metabolites were, in decreasing order, N-Acetyl-S-(3,4-Dihydroxybutyl)-L-Cysteine (DHB) + N-Acetyl-S-(4-hydroxy-2-butenyl)-L-Cysteine (MB3) (butadiene metabolites), N-Acetyl-S-(2-Carboxyethyl)-L-Cysteine (CEM) and N-Acetyl-S-(3-Hydroxypropyl)-L-Cysteine (HPM) (urinary acrolein metabolites), and 2-Methylhippuric acid (2 MH) and 3-Methylhippuric acid and 4-Methylhippuric acid (34 M) (urinary xylene metabolites). These were followed by N-Acetyl-S-(3-hydroxypropyl-1-methyl)-L-Cysteine (PMM), 2-Aminothiazoline-4-carboxylic acid (ATC), Phenylglyoxylic acid (PHG), N-Acetyl-S-(2-carbamoylethyl)-L-Cysteine (AAM) + N-Acetyl-S-(N-methylcarbamoyl)-L-Cysteine (AMC) (acrylamide metabolites). The remaining VOC metabolites were mandelic acid (MAD), N-Acetyl-S-(2-hydroxypropyl)-L-Cysteine (HP2), N-Acetyl-S-(benzyl)-L-Cysteine (BMA), N-Acetyl-S-(n-propyl)-L-Cysteine (BPM), and N-Acetyl-S-(2-cyanoethyl)-L-Cysteine (CYM) (Table 2).

Details on the distribution of creatinine-corrected urinary VOC metabolites levels by the characteristics of study participants are reported in Table 2. Briefly, urinary VOCs metabolites were higher in children aged 6 to 11 years versus 12 to 17 years old for the markers of acrolein, 1,3-butadiene, cyanide, toluene, styrene, ethylbenzene/styrene, and crotonaldehyde. They were higher in females versus males for markers of cyanide. They were highest in non-Hispanic White than other participants for the biomarkers of acrylamide, 1,3-butadiene, acrylonitrile, and ethylbenzene/styrene. Biomarkers of ethylbenzene/styrene were higher in participants with PIR > versus ≤ 1, whereas biomarkers of acrylamide and acrylonitrile were higher in those with serum cotinine ≥ versus <1 ng/mL. Participants with normal versus overweight or obese BMI had highest levels of biomarkers of xylene, acrylamide, acrolein, 1,3-butadiene, cyanide, ethylbenzene/styrene, and crotonaldehyde.

In Supplemental Table S1, we reported FEV_1_ % predicted and the FEV_1_/FVC ratio per characteristics of study participants and found that FEV_1_/FVC was higher in females than in males and obese versus normal or overweight participants.

### 3.2. Urinary VOC Metabolites Intercorrelation

**Figure 1:**
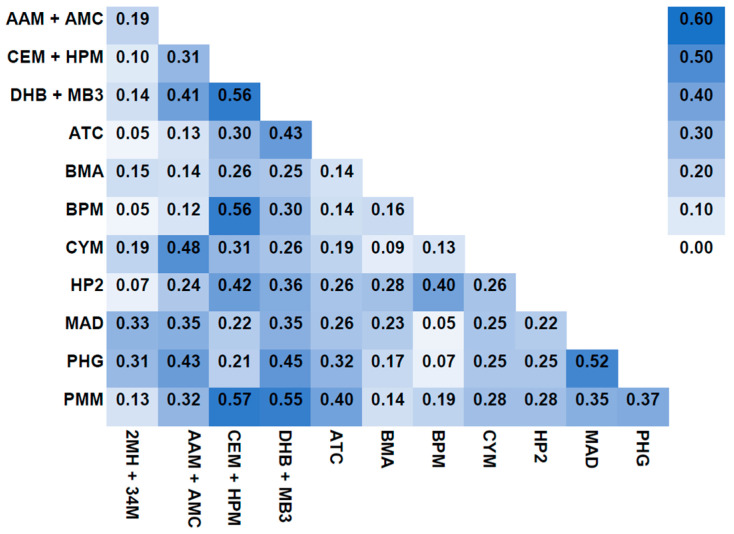
Correlation heatmap and coefficients of creatinine-corrected urinary VOC metabolites.

Creatinine-corrected urinary metabolites of acrolein were strongly correlated with those of 1,3-butadiene (r: 0.56), 1-bromopropane (r: 0.56) and crotonaldehyde (r: 0.57). 1,3-butadiene urinary metabolites were strongly correlated with urinary metabolites of crotonaldehyde (r: 0.56) (Figure 1).

### 3.3. VOC Urinary Metabolites and Lung Function

**Figure 2:**
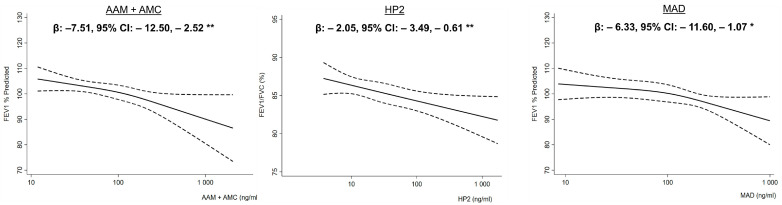
Restricted cubic splines (solid lines) and their corresponding 95% confidence interval (dashed lines) for the associations of acrylamide (AAM + AMC) and styrene (MAD) urinary metabolites with FEV_1_ % predicted and of propylene oxide (HP2) urinary metabolite with FEV_1_/FVC. Models adjusted for age, sex, race/ethnicity, the poverty income ratio, body mass index, height, log-transformed serum cotinine, and urinary creatinine. * = *p* < 0.05, ** = *p* < 0.01.

Higher urinary metabolites for acrylamide (β: −7.95, 95% CI: −13.69, −2.21) and styrene levels (β: −6.33, 95% CI: −11.60, −1.07) were associated with lower FEV_1_ % predicted, while higher propylene oxide urinary metabolite concentrations were associated with lower FEV_1_/FVC % (β: −2.05, 95% CI: −3.49, −0.61) (Table 3). The restricted cubic splines for the associations of urinary metabolites for acrylamide and styrene with FEV_1_ % predicted and as well as of urinary metabolites and propylene oxide with FEV_1_/FVC % are reported in Figure 2 and show no evidence of a threshold effect.

**Figure 3:**
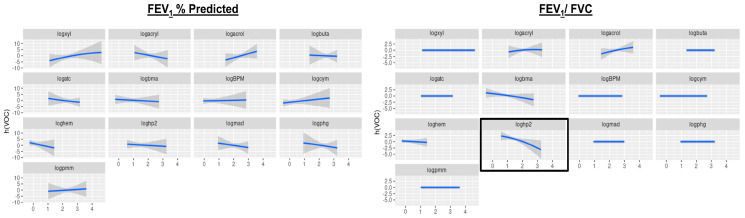
Univariate exposure-outcome relationship for each VOC metabolites association with FEV_1_ % predicted and FEV_1_/FVC, with all other VOC metabolites fixed at their median value. Bayesian Kernel Machine Regression models adjusted for age, sex, race/ethnicity, the poverty income ratio, body mass index, height, log-transformed serum cotinine, and urinary creatinine. Rectangle indicates a significant association. The blue line indicate the cubic splines and the shadows indicate the 95% confidence intervals.

In BKMR modeling, we found only the urinary metabolite for propylene oxide to be associated with low FEV_1_/FVC (Figure 3)

In effect modification testing, the association of 1-bromopropane urinary metabolite with lower FEV_1_ % predicted was modified by serum cotinine (P_interaction_: 0.04) and a relationship was observed in participants with serum cotinine >1 ng/mL (suggestive of exposure to secondhand smoke [SHS]) (β: −6.26, 95% CI: −9.69, −2.82), but not in those with serum cotinine ≤1 ng/mL. The association of crotonaldehyde urinary metabolite with lower FEV_1_ % predicted was modified by obesity (P_interaction_ < 0.001) and a relationship was observed in obese or overweight participants (β: −15.42, 95% CI: −26.76, −4.08), but not in those with normal body weight (Table 4). The associations of urinary VOC metabolites with FEV_1_ % predicted did not vary by the presence of pre-existing asthma and the associations of the urinary metabolites with FEV_1_/FVC % did not differ by the presence of pre-existing asthma, obesity, and serum cotinine. Though the interactions were not significant, we observed trends towards more significant associations of acrylamide, 1-butadiene, styrene, ethylbenzene, and crotonaldehyde with lower FEV_1_ % predicted in overweight or obese participants.

## 4. Discussion

This analysis of a U.S. representative sample is the first study on the associations of biomarkers of VOC exposure and lung function in children and adolescents. It suggests that urinary metabolites for acrylamide, propylene oxide and styrene are associated with impaired lung function in children and adolescents. Moreover, urinary metabolites of 1-bromopropane in those with biochemically validated SHS exposure levels and urinary metabolites of crotonaldehyde in overweight/obese participants were associated with lower FEV_1_ % predicted.

### 4.1. Acrylamide and Lower FEV1 % Predicted

Acrylamide is one of the most common VOCs found in food that can emanate from the heating of starchy foods (e.g., potatoes, grains) at temperatures ≥ 120 °C, making human exposure to this chemical common [18]. Although food has been reported to be the main source of acrylamide exposure, the chemical is also found in water and sewage treatment products, cosmetics, plastics, glue, or soap, and can be found in tobacco smoke [19]. In the body, acrylamide is conjugated with glutathione, causing oxidative stress, a potential mechanism for reduced lung function associated with this exposure [19].

Our present analysis is the first study on biomarkers of acrylamide exposure and lung function in children and adolescents. Prior related research that did not use biomarkers includes the Norwegian Mother and Child Cohort Study (MoBa) that estimated prenatal dietary exposure to acrylamide using a food questionnaire and to examine its relation with wheeze within the first year of life [19]. The study had a major limitation of not including biomarkers of acrylamide exposure and found no association between questionnaire-estimated dietary acrylamide exposure and wheezing [19]. In non-occupational general populations, only one study that included biomarkers of acrylamide exposure examined its relationship with lung function. It included 3271 Chinese adults with urinary measurement of acrylamide metabolites which were found to be associated with lower FEV_1_ and FVC [20]. The study also reported that inflammation, assessed using C-reactive protein, mediated these associations and therefore, was a potential mechanism [20].

### 4.2. Propylene Oxide and Lower FEV_1_/FVC

Propylene oxide is a highly volatile chemical used in polyethers for polyurethane foams, in propylene glycol and other polyglycols, as well as in soil sterilizers; for the past 15 years, it has also been used as a food fumigant to reduce bacteria, mold and yeast in dried fruits and nuts in the U.S. [21]. Humans are exposed to propylene oxide mainly through inhalation; however, exposure by foods contaminated with the chemical is also possible [21]. Consistent with our findings and as potential mechanisms for the association with reduced respiratory function, propylene oxide is well known to cause respiratory irritation as well as oxidative stress and inflammation due to glutathione depletion from conjugation catalyzed by glutathione S-transferase and epoxide hydrolase [22]. In adults from the general population, urinary propylene oxide metabolite was suggested to be associated with lower FEV_1_ % predicted [8]. Although we found no previous study on biomarkers of propylene oxide exposure and lung function in children and adolescents, urinary metabolites of this chemical have been found to be associated with asthma prevalence in a sample of 321 participants [1]. The study also proposed that the association may be mediated by oxidative DNA damage [1].

### 4.3. Styrene and Lower FEV_1_ % Predicted

Styrene is a chemical used in plastic and resins to which humans are exposed through inhalation and ingestion of contaminated food [23,24]. It is purportedly associated with irritation of mucous membranes and respiratory symptoms, especially in occupational settings characterized by high exposure to the chemical [25]. In experimental animal models, exposure to styrene also promotes the production of interferon γ and allergic sensitization by upregulating the activity of antigen-presenting cells to cause eosinophilic inflammation and subsequent airway hyperreactivity [26]. In children, the literature on styrene exposure and respiratory outcomes is limited to symptoms and has not included pulmonary function measures in children. In the Leipzig Allergy High-Risk Children Study (LARS), styrene measured for 4 weeks after birth in infants’ bedrooms using passive sampling was associated with increased risks of respiratory infections but not wheeze at six weeks after birth [26]. Consistent with our findings in the current study, we previously reported that urinary metabolites of styrene to be associated with a lower FEV_1_/FVC ratio among adult NHANES participants [8].

### 4.4. 1-Bromopropane, Crotonaldehyde and Effect Modification by Serum Cotinine and Obesity

1-bromopropane is a solvent in adhesive, degreasing, cleaning, and automobile care products and can also be used as an intermediate in pharmaceuticals and insecticides production; it enters the human body through inhalation and dermal contact, and to a lesser extent, through digestion [27,28]. 1-Bromopropane causes oxidative stress and oxidative damage via glutathione depletion through conjugation as well as via downregulation of glutathione peroxidase expression [27,29]. Yet, we found the association between urinary 1-bromopropane metabolite and FEV_1_ % predicted only in participants with cotinine levels indicative of SHS exposure. Prior epidemiological studies conducted on occupational settings with high 1-bromopropane exposure levels reported associations with adverse respiratory outcomes [27]. In one of the rare studies on 1-bromopropane and respiratory outcomes conducted in general populations, urinary metabolite levels of this chemical were associated with reduced FEV_1_ and FVC volumes among 3259 Chinese adults [30]. This association was observed in both smokers and non-smokers and was partially mediated by oxidative DNA damage [30]. The mechanisms for effect modification by smoking on 1-bromopropane and lung function are unclear.

Crotonaldehyde is used in sorbic acid, leather tanning, colorants, pesticides, and pharmaceuticals that can be found in tobacco smoke as well as in exhaust fumes and is a potent mucous irritant [31]. Acute occupational exposure to crotonaldehyde may cause respiratory distress syndrome in humans [32]. In animal models, crotonaldehyde induces edema, hyperemia, cell necrosis through inflammation and oxidative stress as well as lung immunological dysfunctions [33,34,35]. Nevertheless, studies on crotonaldehydes and respiratory outcomes in general populations are lacking and there has been no investigation on children’s lung function. Although the exact mechanism for effect modification by overweight or obesity on the association between crotonaldehyde and lower lung function is unclear, this association has been observed with other environmental exposures such as nitrogen dioxide and potential mechanistic hypotheses have been formulated [36]. It is possible that obese patients may have higher respiratory rates leading to greater intake and lung deposition of inhaled exposures [37]. Obesity and VOCs may also have potentiation effects on inflammation and oxidative stress in affecting respiratory epithelium and smooth muscle cells [36]. Moreover, diet and altered gut microbiome may influence the response of the immune system and the airways to environmental exposures [38].

### 4.5. Limitations and Strengths

This analysis has limitations. The temporality between exposure to VOCs and lung function is unknown and causality cannot be established because of the cross-sectional nature of NHANES. VOC urinary metabolites were measured only in a single sample of spot urine and may not reflect long-term exposure. Data on factors that might have affected urinary VOCs metabolites such as diet and other covariates such as physical activity and other environmental exposures potentially associated with respiratory outcomes were not available and could not be adjusted for [39,40]. Data on urinary VOC metabolites for more recent years was available, but data from both spirometry and measurement of urinary VOC metabolites were only available in the NHANES 2011–2012 cycle. However, our analysis has major strengths. It includes a sample representative of the population of children and adults in the U.S., which makes the findings more generalizable. Urinary VOC metabolites have a half-life in the order of days, which is longer than that of blood VOCs which is only of hours. Urinary VOC metabolites also indicate VOC exposure from indoor and outdoor sources and are a better indicator of internal dose than environmental indoor VOCs measurement [41,42,43,44]. Exposure measures were performed by laboratories of the National Center of Environmental Health (NCEH) of the CDC with rigorous methods and several covariates were adjusted for in our analysis to minimize residual confounding. Notably, our analysis is the first to examine the association of biomarkers of VOC exposure with lung function impairment in children and adolescents.

## 5. Conclusions

This analysis of cross-sectional data from a sample representative of the U.S. child and adolescent population found reduced lung function with urinary VOC metabolites for acrylamide, propylene oxide, styrene, 1-bromopropane (in participants exposed to SHS), and crotonaldehyde (in overweight or obese participants). However, prospective studies with repeated measures of exposure are needed to confirm these findings and to investigate their underlying mechanisms.

## Figures and Tables

**Table 1:** Description and detection of VOC urinary metabolites, NHANES 2011–2012 (*N* = 505).

Urinary Metabolites	Abbreviations	Parent Compounds	LOD (ng/mL)	Detection %
2-Methylhippuric acid	2 MH	Xylene	5.00	95.0
3-Methylhippuric acid and 4-Methylhippuric acid	34 M	Xylene	8.00	100.0
N-Acetyl-S-(2-carbamoylethyl)-L-Cysteine	AAM	Acrylamide	2.20	100.0
N-Acetyl-S-(N-methylcarbamoyl)-L-Cysteine	AMC	Acrylamide	6.26	99.8
N-Acetyl-S-(2-Carboxyethyl)-L-Cysteine	CEM	Acrolein	6.96	98.0
N-Acetyl-S-(3-Hydroxypropyl)-L-Cysteine	HPM	Acrolein	13.00	100.0
N-Acetyl-S- (3,4-Dihydroxybutyl)-L-Cysteine	DHB	1,3-Butadiene	5.25	100.0
N-Acetyl-S-(4-hydroxy-2-butenyl)-L-Cysteine	MB3	1,3-Butadiene	0.60	95.4
2-Aminothiazoline-4-carboxylic acid	ATC	Cyanide	15.00	96.5
N-Acetyl-S-(benzyl)-L-Cysteine	BMA	Toluene	0.50	99.7
N-Acetyl-S-(n-propyl)-L-Cysteine	BPM	1-Bromopropane	1.20	64.5
N-Acetyl-S-(2-cyanoethyl)-L-Cysteine	CYM	Acrylonitrile	0.50	91.7
N-Acetyl-S-(2-hydroxypropyl)-L-Cysteine	HP2	Propylene oxide	5.30	93.0
Mandelic acid	MAD	Styrene	12.00	99.0
Phenylglyoxylic acid	PHG	Ethylbenzene, styrene	12.00	99.5
N-Acetyl-S-(3-hydroxypropyl-1-methyl)-L-Cysteine	PMM	Crotonaldehyde	1.13	100.0

LOD: level of detection.

**Table 2:** Geometric means (GMs [SE]) of creatinine-corrected urinary VOCs by characteristics of study participants, NHANES 2011–2012 (*N* = 505).

Characteristics	%	VOC Urinary Metabolites (ng/g Creatinine)											
		2 MH + 34 M	AAM + AMC	CEM + HPM	DHB + MB3	ATC	BMA	BPM	CYM	HP2	MAD	PHG	PMM
All	100	251.06 (15.62)	142.44 (4.91)	302.93 (14.05)	316.24 (9.88)	204.29 (10.49)	7.52 (0.33)	3.29 (0.37)	2.10 (0.14)	29.68 (1.54)	116.66 (4.13)	196.57 (5.25)	208.32 (7.73)
Age groups													
6 to 11 years old	47.0	248.22 (16.25)	149.40 (2.48)	**344.57 (11.04)**	**381.32 (14.66)**	**344.03 (16.62)**	**8.86 (0.41)**	3.56 (0.23)	2.15 (0.11)	25.56 (1.97)	**130.51 (5.48)**	**230.28 (5.67)**	**249.06 (10.76)**
12 to 17 years old	53.0	253.60 (19.74)	136.54 (8.84)	**270.21 (20.68)**	**267.84 (8.90)**	**128.62 (9.08)**	**6.51 (0.51)**	3.04 (0.56)	2.06 (0.21)	25.59 (1.69)	**105.60 (6.60)**	**170.81 (7.78)**	**177.78 (11.05)**
Sex													
Males	50.1	248.98 (23.69)	138.56 (4.59)	296.21 (9.32)	321.67 (9.05)	**176.51 (15.09)**	7.11 (0.49)	3.04 (0.29)	1.90 (0.14)	29.08 (1.86)	113.81 (3.96)	188.73 (4.84)	198.40 (8.43)
Females	49.9	253.16 (17.10)	146.45 (7.47)	309.84 (26.36)	310.89 (15.16)	**236.58 (14.76)**	7.96 (0.39)	3.55 (0.66)	2.33 (0.27)	30.30 (2.09)	119.59 (5.52)	204.77 (7.52)	218.79 (13.35)
Race/ethnicity													
Non-Hispanic White	54.1	276.90 (25.78)	**160.52 (7.26)**	294.33 (23.38)	**337.73 (18.89)**	207.98 (15.22)	7.06 (0.49)	3.04 (0.57)	**2.27 (0.24)**	29.56 (2.61)	118.20 (5.34)	**224.78 (5.67)**	216.79 (11.92)
Non-Hispanic Black	16.0	255.52 (12.18)	**115.69 (5.87)**	305.79 (17.95)	**267.06 (9.68)**	165.80 (17.95)	9.33 (0.88)	3.57 (0.38)	**2.25 (0.22)**	28.65 (2.14)	106.92 (6.33)	**160.02 (5.29)**	177.40 (10.71)
Mexican American	16.0	225.52 (12.84)	**134.52 (3.11)**	309.46 (19.06)	**308.42 (13.70)**	226.05 (30.95)	7.64 (0.56)	3.52 (0.63)	**1.64 (0.05)**	32.79 (1.93)	124.82 (5.37)	**177.33 (4.97)**	212.26 (16.87)
Other	13.9	190.06 (13.73)	**121.40 (3.40)**	327.16 (20.67)	**306.12 (8.94)**	215.64 (26.23)	7.37 (0.56)	3.75 (0.45)	**1.92 (0.17)**	28.03 (1.50)	113.34 (5.69)	**166.37 (5.64)**	210.06 (14.74)
PIR													
≤1	24.0	230.12 (18.45)	136.42 (6.25)	305.64 (17.76)	308.81 (14.43)	202.84 (19.26)	8.06 (0.59)	3.75 (0.41)	2.34 (0.26)	29.11 (2.11)	111.25 (5.47)	**178.49 (8.81)**	205.00 (12.71)
>1	76.0	258.06 (18.09)	144.40 (5.69)	302.08 (17.21)	318.63 (10.69)	204.75 (12.81)	7.36 (0.35)	3.16 (0.39)	2.03 (0.13)	29.86 (1.66)	118.42 (4.60)	**202.65 (4.78)**	209.38 (10.48)
Cotinine													
<1.0 ng/mL	88.6	241.54 (18.03)	**138.06 (4.86)**	297.44 (13.65)	312.44 (8.66)	203.33 (12.47)	7.65 (0.37)	3.31 (0.40)	**1.75 (0.09)**	28.73 (1.46)	115.30 (4.77)	193.89 (5.24)	204.00 (7.73)
≥1.0 ng/mL	11.4	338.52 (42.90)	**181.37 (16.79)**	349.00 (31.79)	347.30 (32.20)	211.90 (34.49)	6.63 (0.77)	3.10 (0.54)	**8.72 (1.62)**	38.21 (5.32)	127.72 (8.05)	218.58 (15.14)	245.03 (24.32)
BMI													
Normal	78.9	**265.95 (17.56)**	**147.20 (5.57)**	**315.01 (15.11)**	**330.45 (10.60)**	**231.39 (13.89)**	7.78 (0.28)	3.48 (0.42)	2.05 (0.14)	31.15 (1.85)	119.05 (4.91)	**208.70 (5.32)**	**221.69 (9.96)**
Overweight	12.5	**217.87 (41.61)**	**134.60 (12.14)**	**264.28 (21.65)**	**280.23 (17.66)**	**117.48 (20.39)**	5.96 (0.72)	2.62 (0.51)	2.31 (0.35)	22.86 (1.90)	111.03 (10.76)	**161.31 (12.20)**	**168.63 (11.36)**
Obese	8.6	**181.82 (18.03)**	**114.36 (10.07)**	**258.18 (18.71)**	**251.96 (10.85)**	**145.92 (15.86)**	7.78 (1.04)	2.69 (0.62)	2.35 (0.46)	27.93 (3.89)	104.06 (10.21)	**151.35 (14.05)**	**160.16 (9.23)**

PIR: poverty income ratio; BMI: body mass index. Bold fonts indicate significant differences in the concentrations of urinary VOC metabolites levels.

**Table 3:** Association of urinary VOCs with FEV_1_ % predicted and FEV_1_/FVC %.

Urinary Metabolites	Parent VOC	FEV_1_ % Predicted	FEV_1_/FVC
2 MH + 34 M	Xylene	0.44 (−2.33, 3.21)	0.11 (−1.60, 1.83)
AAM + AMC	Acrylamide	−7.95 (−13.69, −2.21) **	−2.25 (−6.08, 1.57)
CEM + HPM	Acrolein	2.06 (−3.73, 7.85)	−1.70 (−4.06, 0.67)
DHB + MB3	1, 3-Butadiene	−3.94 (−11.37, 3.50)	0.82 (−4.65, 3.01)
ATC	Cyanide	1.38 (−3.09, 5.86)	−0.74 (−3.06, 1.58)
BMA	Toluene	−2.93 (−9.82, 3.96)	−1.22 (−3.53, 1.09)
BPM	1-Bromopropane	2.20 (−2.58, 6.97)	−0.70 (−1.95, 0.54)
CYM	Acrylonitrile	0.53 (−6.14, 7.21)	−1.21 (−3.13, 0.71)
HP2	Propylene oxide	−1.74 (−6.92, 3.43)	−2.05 (−3.49, −0.61) **
MAD	Styrene	−6.33 (−11.60, −1.07) *	0.56 (−1.74, 2.87)
PHG	Ethylbenzene, styrene	−3.33 (−10.09, 3.43)	−1.83 (−4.35, 0.69)
PMM	Crotonaldehyde	1.36 (−3.32, 6.05)	−1.17 (−4.34, 2.01)

Values reported in the table are regression coefficients (β) with corresponding 95% confidence intervals. * = *p* < 0.05, ** = *p* < 0.01. Models adjusted for age, sex, race/ethnicity, the poverty income ratio, body mass index, height, log-transformed serum cotinine, and urinary creatinine. Bold font indicates significant associations.

**Table 4:** Effect modification of asthma, obesity, and serum cotinine on association of urinary VOC metabolites with FEV_1_ % predicted.

Urinary Metabolites	Parent VOC	Obesity			Serum Cotinine		
		No	Yes	P_interaction_	≤1 ng/mL	>1 ng/mL	P_interaction_
2 MH + 34 M	Xylene	1.25 (−2.42, 4.93)	−2.40 (−8.78, 3.99)	0.48	−0.27 (−2.8, 2.04)	6.99 (−0.43, 14.41)	0.21
AAM + AMC	Acrylamide	−4.92 (−11.78, 1.94)	−15.40 (−23.56, −7.24) ***	0.90	−7.28 (−11.78, −2.79) **	−7.55 (−33.77, 18.68)	0.57
CEM + HPM	Acrolein	4.92 (−2.04, 11.87)	−1.54 (−14.15, 9.07)	0.78	3.10 (−3.10, 9.30)	5.21 (−8.63, 19.05)	0.69
DHB + MB3	1,3 Butadiene	0.75 (−8.58, 10.92)	−16.61 (−28.80, −4.41) *	0.70	−4.26 (−12.86, 4.33)	8.37 (−9.97, 26.72)	0.31
ATC	Cyanide	1.97 (−2.24, 6.19)	2.03 (−5.40, 9.45)	0.99	1.20 (−2.87, 5.28)	4.98 (−12.20, 22.17)	0.88
BMA	Toluene	−1.75 (−10.19, 6.70)	−4.58 (−13.44, 4.28)	0.66	−2.38 (−8.20, 3.45)	−0.81 (−16.92, 15.29)	0.66
BPM	1-Bromopropane	4.54 (−1.14, 10.23)	−1.49 (−5.38, 2.29)	0.15	4.29 (−0.15, 8.73)	−6.26 (−9.69, −2.82) **	**0.04**
CYM	Acrylonitrile	0.88 (−6.70, 8.45)	1.54 (−8.88, 11.95)	0.82	0.81 (−5.93, 7.56)	−2.62 (−21.65, 16.40)	0.56
HP2	Propylene oxide	0.25 (−5.35, 5.86)	−7.36 (−17.73, 3.01)	0.76	−0.38 (−6.26, 5.49)	−11.70 (−31.40, 8.00)	0.72
MAD	Styrene	−4.97 (−11.66, 3.72)	−14.93 (−25.66, −4.20) **	0.93	−5.38 (−11.02, 0.25)	−16.17 (−34.09, 1.75)	0.72
PHG	Ethylbenzene, styrene	−0.61 (−10.22, 9.01)	−10.42 (−20.77, −0.06) *	0.92	−2.61 (−9.29, 4.06)	−8.66 (−42.78, 25.45)	0.80
PMM	Crotonaldehyde	4.96 (−0.80, 10.72)	−15.42 (−26.76, −4.08) *	**<0.001**	2.07 (−4.20, 8.33)	3.84 (−12.90, 20.57)	0.67

* = *p* < 0.05, ** = *p* < 0.01, *** = *p* < 0.001. Values reported in the table are regression coefficients (β) with corresponding 95% confidence intervals. Models adjusted for age, sex, race/ethnicity, the poverty income ratio, body mass index, height, log-transformed serum cotinine, and urinary creatinine. BMI or serum cotinine were not adjusted when stratified by these variables. Gray shading indicates significant effect modification and bold font designates significant associations.

## Data Availability

The dataset was derived from sources in the public domain provided by the National Center for Health Statistics: https://wwwn.cdc.gov/nchs/nhanes/Default.aspx (accessed on 24 November 2023).

## Supplementary Materials

The following supporting information can be downloaded at: https://www.mdpi.com/article/10.3390/toxics12040289/s1, Table S1: Geometric means (GMs [SE]) of FEV_1_ % predicted and FEV_1_/FVC by characteristics of study participants, NHANES 2011–2012 (*N* = 505).
