# Supplementary material for: Urinary Volatile Organic Compound Metabolites Are Associated with Reduced Lung Function in U.S. Children and Adolescents
Source: Toxics. 2024 Apr 16;12(4):289. doi: 10.3390/toxics12040289 (PMC11054577; doi:10.3390/toxics12040289)
Supplement: Supplementary file 1 [file toxics-12-00289-s001.zip › toxics-2939233-supplementary.pdf]

Supplemental Table S1: Geometric means (GMs [SE]) of FEV_1 % predicted and FEV_1/FVC by characteristics of study participants, NHANES 2011-2012 (N=505)

characteristics	FEV_1 % predicted	FEV_1/FVC
All	98.07 (1.21)	85.25 (0.51)
Age groups		
6 to 11 years old	99.56 (1.59)	85.08 (0.61)
12 to 17 years old	96.79 (1.54)	85.41 (0.77)
Sex		
Males	97.48 (1.57)	**83.34 (0.77)**
Females	98.68 (1.65)	**87.22 (0.63)**
Race/ethnicity		
Non-Hispanic White	97.31 (1.89)	84.45 (0.80)
Non-Hispanic Black	100.60 (2.07)	85.50 (0.48)
Mexican American	98.16 (0.83)	85.92 (0.53)
Other	-	87.36 (0.61)
PIR		
≤ 1	98.28 (0.95)	85.75 (0.62)
> 1	98.01 (1.53)	85.09 (0.65)
Cotinine		
<1.0 ng/mL	101.04 (1.39)	85.41 (0.79)
≥ 1.0 ng/mL	97.68 (1.47)	84.04 (0.55)
BMI		
Normal	97.27 (1.29)	**85.44 (0.58)**
Overweight	102.18 (2.41)	**85.81 (0.95)**
Obese	100.23 (2.74)	**82.71 (0.99)**

Abbreviations: PIR: poverty income ratio; BMI: body mass index.
Bold fonts indicate significant differences in lung function.
